# Case Report: A rare case of breast leiomyoma with literature review

**DOI:** 10.3389/fonc.2026.1678179

**Published:** 2026-02-06

**Authors:** Xiao Feng, Shue Zeng

**Affiliations:** 1 Department of Ultrasound, Hubei Cancer Hospital, Tongji Medical College, Huazhong University of Science and Technology, Wuhan, China; 2 Breast Cancer Center, Hubei Cancer Hospital, National Key Clinical Specialty Discipline Construction Program, Hubei Provincial Clinical Research Center for Breast Cancer, Wuhan, China

**Keywords:** breast leiomyoma, breast tumor, contrast-enhanced ultrasound, lelastography, ultrasound

## Abstract

Breast leiomyoma is a rare benign mesenchymal tumor that typically manifests as a solitary, slow-growing mass. This article presents a case of a 58-year-old female with a history of follicular lymphoma confirmed by pathological examination of resected lymph nodes in November 2019. Follow-up positron emission tomography/computed tomography (PET-CT) revealed a breast soft-tissue mass initially classified as likely benign. Subsequent chest computed tomography (CT) in May 2024 demonstrated enlargement of the lesion, raising suspicion of lymphomatous infiltration. The mammogram shows an irregular mass shadow with shallow lobulation and scattered punctate and coarse calcification shadows. It is classified as BI-RADS 4b. Multimodal ultrasound evaluation (including grayscale ultrasound, color doppler, shear-wave elastography, strain elastography, and contrast-enhanced ultrasound) identified a solid breast mass categorized as BI-RADS 4b. Ultrasound-guided core needle biopsy revealed adenosis with focal fibroadenomatoid changes. Definitive diagnosis was achieved through surgical excision, with histopathology demonstrating fascicular arrangements of spindle-shaped smooth muscle cells on hematoxylin-eosin staining, corroborated by immunohistochemical confirmation (SMA+/desmin+). Postoperative surveillance at 6 months showed no recurrence. A search was conducted in the PubMed database using “breast leiomyoma” as the keyword, with the publication date limited to January 1, 2015, to May 3, 2025. A total of 192 articles were retrieved. After applying rigorous selection criteria, 11 case reports that met the criteria were finally included for systematic review, involving a total of 12 cases of pathologically confirmed breast leiomyoma (one report contained 2 cases). Notably, only one study comprehensively characterized the contrast-enhanced ultrasound features, and no elastography examination was performed in any of the cases. This report presents a case of breast leiomyoma in a patient with a history of lymphoma. The imaging features were highly suggestive of malignancy, yet histopathological examination confirmed a benign lesion. This study aims to analyze the clinical and preoperative imaging characteristics of this case. Combined with a comprehensive literature review, it thoroughly explores the diagnostic imaging manifestations of breast leiomyoma, providing a reference for clinical differential diagnosis.

## Introduction

Breast leiomyoma, recognized as one of the rarest mesenchymal tumors of the breast, accounts for less than 1% of all breast neoplasms (1, 2). Since its first report by Strong et al. in 1913 (3), the cumulative number of cases in the global literature remains extremely limited. Epidemiological data show that the disease is prevalent in middle-aged women (mean age 47.6 years) (4), with a characteristic anatomical distribution: approximately 80% occur in superficial subareolar areas. At the same time, deep breast parenchyma originates in only a minority (5, 6). Despite its benign biological behaviour, presenting as a slow-growing, isolated mass (7, 8), preoperative imaging for differential diagnosis is challenging, and experience with novel techniques such as elastography and contrast-enhanced ultrasound (CEUS), especially in such cases, remains extremely scarce. In this paper, we report a case of lymphoma combined with breast leiomyoma and summarize the previous literature on imaging of breast smooth muscle tumors. In this study, we provide a rare case of this type, expecting it to benefit subsequent research.

## Case history

**Figure 1:**
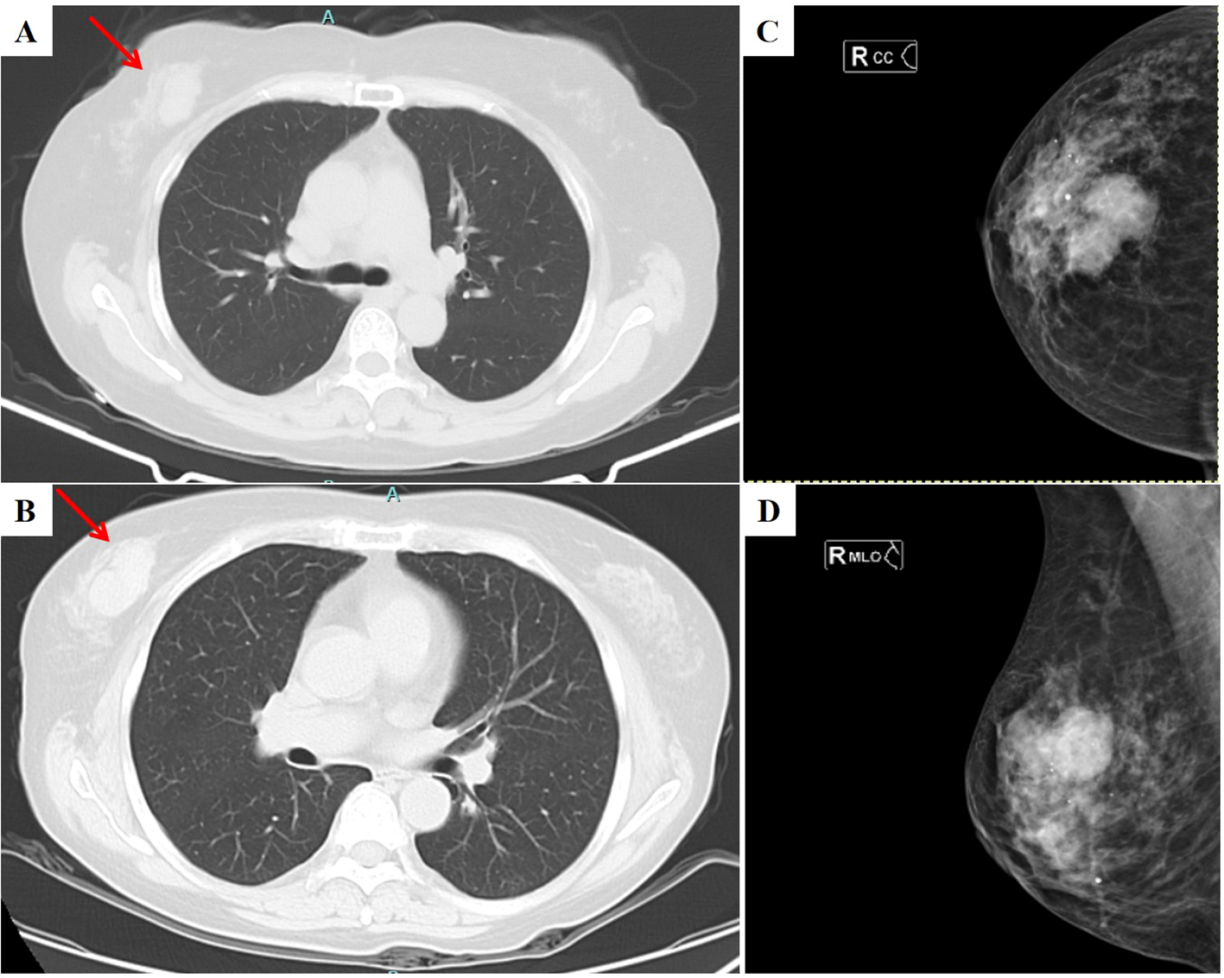
CT and MMG images of the tumor. **(A)** Chest CT showed an irregular solid mass in the upper quadrant of the right breast (this image was taken on October 26, 2023. The location of the lesion is indicated by the red arrow); **(B)** Chest CT showed an irregular solid mass in the upper quadrant of the right breast that was larger than before (this image was taken on May 29, 2024. The location of the lesion is indicated by the red arrow); **(C)** The head and tail positions of the MMG show an irregular mass shadow in the middle and upper part of the right breast (this image was taken on June 11, 2024); **(D)** The internal and external oblique position of MMG shows an irregular mass shadow in the middle and upper part of the right breast (this image was taken on June 11, 2024); CT, computed tomography; MMG, mammography.

The patient is a 58-year-old woman, who presented in November 2019 with “multiple painless masses in the neck for more than 1 year”, underwent left submandibular lymph node dissection, and was pathologically diagnosed with follicular lymphoma (grade 1), with immunohistochemistry showed that CD20 positive, CD10 positive, bcl2 positive, bcl6 positive and the Ki67 index was approximately 8%. Positron emission tomography/computed tomography (PET-CT) showed enlarged lymph nodes in multiple areas (bilateral submandibular, axillary, retroperitoneal, etc.) with suspected infiltration of the liver and bone, and a soft-tissue mass in the right breast (tendency toward benign). Bone marrow biopsy confirmed lymphoma bone marrow invasion; no treatment has been given for now. In October 2020, a periosteal puncture was performed due to otitis media. The puncture results indicated disease progression (grade III follicular lymphoma), and the patient was transferred to our hospital in November of the same year. Thoracic, abdominopelvic, and pelvic computed tomography (CT) showed a right breast mass (**Figure 1A**), and lymphoma infiltration was highly suspected on imaging, accompanied by generalized multiple lymph node enlargement and organ involvement (common hepatic artery and mesenteric vascular encompassing). Eight cycles of chemotherapy with R-CHOP (rituximab, cyclophosphamide, doxorubicin, vincristine, and prednisone) were completed from November 2020 to May 2021, and the efficacy was evaluated as stable disease (SD). From May 2021 to May 2024, no special treatment was administered. Regular follow-up examinations showed that the lesions remained stable, indicating that the follicular lymphoma was in a stable phase of the disease.

**Figure 2:**
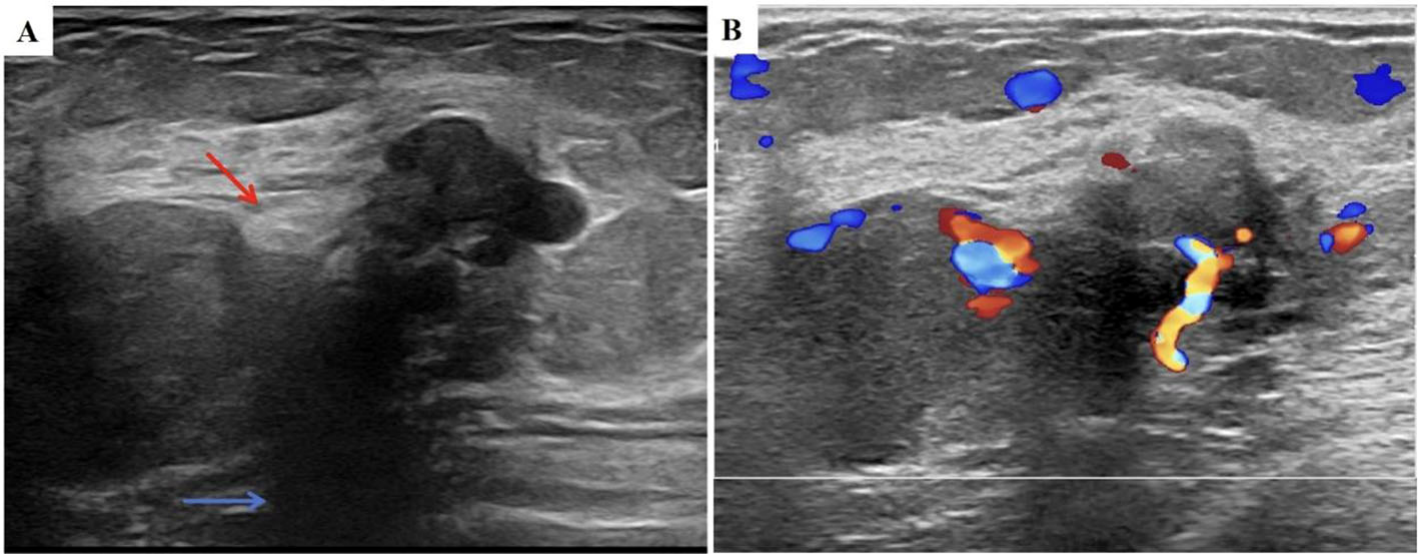
US images of the tumor. **(A)** US of right breast nodule (this image was taken on June 11, 2024. The calcifications is identified by the red arrow, and the acoustic shadowing is marked with the blue arrow); **(B)** Color Doppler image of right breast nodule (this image was taken on June 11, 2024); US, ultrasound; SWE, Shear wave elastography; SE, Strain elastography.

**Figure 3:**
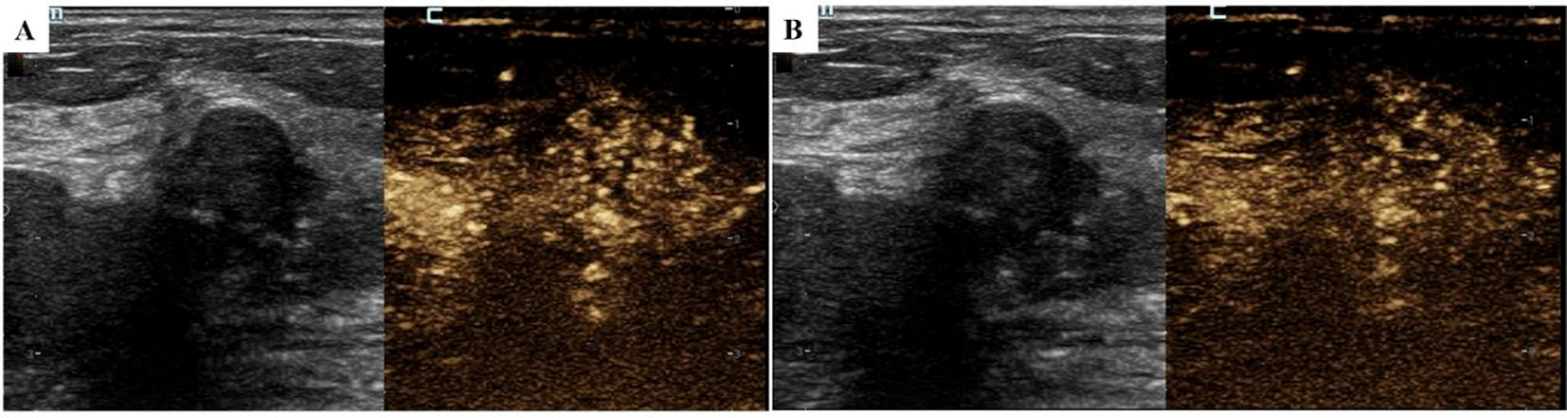
CEUS images of the tumor. **(A)** CEUS arterial phase of right breast nodule (this image was taken on June 11, 2024; the image shows the situation 20 seconds after the contrast agent was injected); **(B)** CEUS venous phase of right breast nodule (this image was taken on June 11, 2024; the image shows the situation 50 seconds after the contrast agent was injected); US, ultrasound; CEUS, contrast-enhanced ultrasound.

**Figure 4:**
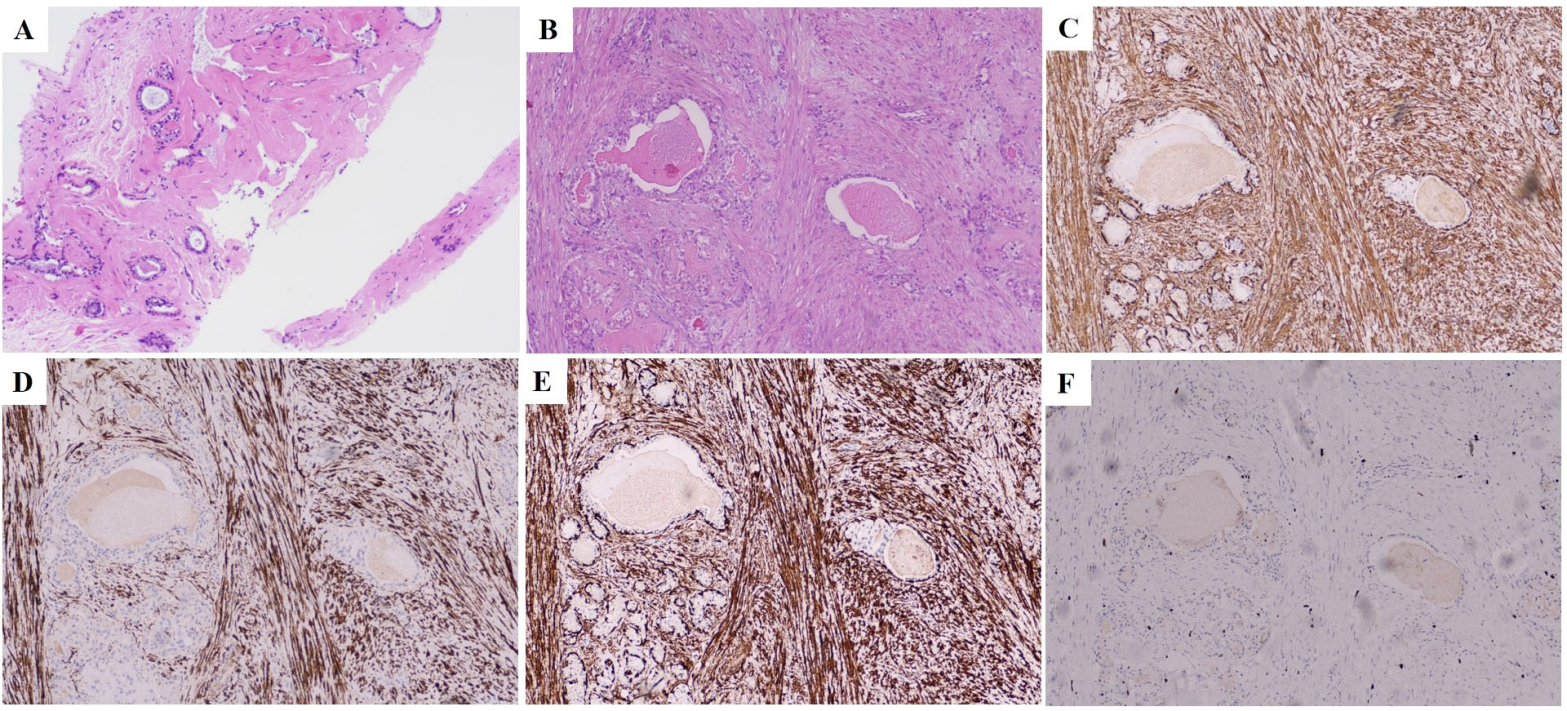
Cell and histopathological features of the tumor. **(A)** A biopsy of the mass in the right breast revealed adenosis with focal fibroadenomatoid changes (H&E, ×100). **(B)** The surgical resection specimen of the right breast mass showed bundled smooth muscle cell (H&E, ×100). **(C)** SMA positive in the right breast mass (IHC staining, ×100; scale bar, 50 µm). **(D)** Desmin positive in the right breast mass (IHC staining, ×100; scale bar, 50 µm). **(E)** Calponin positive in the right breast mass (IHC staining, ×100; scale bar, 50 µm). **(F)** The Ki-67 of the right breast mass was 3% H&E, hematoxylin-eosin; IHC, Immunohistochemistry.

In May 2024, a follow-up chest CT scan revealed an increase in the size of the right breast mass compared to previous imaging (**Figure 1B**). The patient was subsequently referred to our breast center for further evaluation. A preoperative systemic evaluation confirmed that the systemic lymphoma status remained unchanged. Tumor markers were within normal limits (CEA: 0.675 ng/mL [standard <5], CA153: 7.31 U/mL [normal <24], CA125: 6.14 U/mL [normal <35]). Mammography (MMG) demonstrated an irregular mass shadow with relatively straightforward margins and shallow lobulation in the upper middle quadrant of the right breast, exhibiting relatively homogeneous density and scattered punctate/coarse calcifications, classified as BI-RADS 4b (**Figures 1C, D**). Grayscale ultrasound showed a hypoechoic mass measuring 4.52×1.84 cm at the 10 o’clock position in the right breast, with well-defined borders but irregular morphology. The lesion demonstrated heterogeneous internal echogenicity, coarse calcifications, and posterior acoustic shadowing (**Figure 2A**). Colour Doppler Flow Imaging (CDFI) revealed relatively abundant intralesional blood flow signals (**Figure 2B**). Further evaluation with shear-wave elastography (SWE) quantified a maximum elasticity value (Emax) of 137 kPa, while strain elastography (SE) assigned an elasticity score of 4/5, both indicating a relatively firm consistency of the mass. Contrast-enhanced ultrasound (CEUS) employed SonoVue^®^ (sulfur hexafluoride; 4.8 mL bolus via antecubital vein followed by 5 mL saline flush) at a mechanical index of 0.082 in Contrast Pulse Sequencing mode. During quiet breathing to minimize motion artefacts, real-time enhancement (0–90 sec post-injection) was recorded. Enhancement patterns (homogeneity/heterogeneity, centripetal/centrifugal filling, perfusion defects) and time-intensity curve parameters (arrival time [AT], time-to-peak [TTP], peak intensity [PI]) were analyzed. CEUS revealed heterogeneous hyperenhancement in the arterial phase (**Figure 3A**) (AT: 14.4 s, TTP: 28.07 s, PI: 20 dB) without post-contrast lesion enlargement, with enhancement intensity significantly exceeding the adjacent normal breast parenchyma. In the venous phase, the lesion showed only slight enhancement. (**Figure 3B**). Based on Multimodal ultrasonographic characterization, the mass was classified as BI-RADS 4b. Ultrasound-guided core needle biopsy revealed adenosis with focal fibroadenomatoid changes (**Figure 4A**). The pathology confirmed the breast leiomyoma diagnosis after the surgical excision: The general specimen shows that the section is grayish-white and has a hard texture. Hematoxylin-eosin (H-E) staining showed bundled smooth muscle cells and extensive collagen fibre deposition (**Figure 4B**). Immunohistochemistry staining showed positive SMA (**Figure 4C**), Desmin (**Figure 4D**), and Calponin (**Figure 4E**), with a Ki-67 proliferation index of approximately 3% (**Figure 4F**). There was no recurrence in the six-month postoperative ultrasound follow-up.

## Discussion

This study reports a rare case of follicular lymphoma coexisting with breast leiomyoma. The patient underwent preoperative MMG and systematic multimodal ultrasound (including grayscale ultrasound, color doppler, SWE, SE, and CEUS). A definitive preoperative diagnosis could not be established; however, the breast leiomyoma was ultimately confirmed by postoperative pathology. A search was conducted in the PubMed database using “breast leiomyoma” as the keyword, with the publication date limited to January 1, 2015, to May 3, 2025. A total of 192 articles were retrieved. After reading the titles, abstracts, and full texts, and applying the inclusion and exclusion criteria (inclusion: case reports with imaging of breast leiomyoma; exclusion: non-related pathological types, etc.), 11 case reports that met the criteria were finally included for systematic review, involving a total of 12 cases of pathologically confirmed breast leiomyoma (one report contained 2 cases) (**Table 1**), which were as follows: CT (1 case): limited lesion (7); MRI (2 cases): lesion enhancement (16, 17); mammography (7 cases): most of the cases showed a well-defined oval-shaped dense tissue mass (1, 6, 9, 10, 12, 18); ultrasonography (11 cases): mainly showed an oval-shaped well-defined hypoechoic nodule with posterior acoustic shadow, some cases showed iso/hypoechoic features (9, 10, 12–18), of which only one case reported CEUS findings (17). Compared with the findings of this study, the grayscale ultrasound characteristics of breast leiomyomas reported in the literature show some consistency and differences: a clear boundary is consistent with previous reports, but the presence of coarse calcification and irregular shape differs. However, descriptions of colour Doppler ultrasound manifestations in the literature are scarce, and only a few studies have addressed this topic. These typically describe the lesions as having no internal blood flow or sparse flow signals, which contrasts with the relatively abundant blood flow signals observed in our cases.

**Table 1:** Imaging of 12 cases of breast leiomyoma.

Author	Year	Imaging manifestations
Miroslav Granic et al. (9)	2015	MMG: Oval, dense mass with indistinct margins US: Oval hypoechoic homogenous mass without cystic features, with lobulated borders, hyperechoic rim, and posterior shadowing CT/MRI: None
Miroslav Granic et al. (9)	2015	MMG: Extremely dense tissue composition and oval, well-circumscribed dense lesion US: Well-defined, oval, slightly lobulated mass that is isoechoic to hyperechoic compared to the breast parenchyma, with no sign of posterior enhancement CT/MRI: None
Giorge Pereira Sampaio et al. (10)	2016	MMG: Dense, well-defined oval nodule US: Hypoechoic oval nodule with lobulated margins and well-defined borders, with no detectable Doppler flow CT/MRI: None
Mehmet Tolga Kafadar et al. (11)	2017	MMG: Dense, oval, non-calcified lesion in the corresponding region US: None CT/MRI: None
Brandão RG et al. (12)	2017	MMG: Isodense circumscribed oval mass US: Hypoechoic oval mass that was predominantly circumscribed but sometimes showed microlobulated margins, and which was parallel to the breast skin CT/MRI: None
Mehmet Eren Yuksel et al. (13)	2018	MMG: None US: Well-defined hypoechoic mass with posterior acoustic shadow CT/MRI: None
Adil Arrob et al. (7)	2019	MMG: Large hypoechoic tumour US: Large hypoechoic tumour CT: Well-limited, without invasion of the chest wall
Zhong E et al. (14)	2020	MMG: None US: Hypoechoic nodule CT/MRI: None
Cai S et al. (15)	2020	MMG: None US: Well-circumscribed, hypoechoic lesion with posterior acoustic enhancement. The color Doppler showed less blood flow into the tumour and abundant blood flow signals around it CT/MRI: None
Chiorean A et al. (16)	2020	MMG: None US: Oval, circumscribed hypoechoic mass with internal vascularity MRI: Intense, homogeneous enhancement in the mass
Zhang S et al. (17)	2023	MMG: None US: Hypoechoic elliptical nodule with well-defined borders and regular morphology, with ultrasonography suggesting homogeneous hyperenhancement of the tumour in the arterial phase MRI: Hypoechoic oval mass that showed progressive enhancement in the arterial phase and diffuse enhancement in the delayed phase
Raghunath P, et al. (18)	2025	MMG: Well-circumscribed, isodense, or hypodense masses US: Well-circumscribed, ellipsoid-shaped, and hypoechoic CT/MRI: None

Regarding CEUS, only one case reported in 2023 described homogeneous hyperenhancement during the arterial phase; in contrast (17), our cases demonstrated heterogeneous hyperenhancement in the arterial phase without a significant change in enhancement area compared to the pre-contrast scan. The innovative value of this study lies in being the first to report a multimodal ultrasound diagnostic system for breast leiomyoma comprehensively. The elastic imaging characteristics are the first report globally, and the description of the CEUS features is the second document following it. At the same time, it is the first to report a clinical case of coexistence of breast leiomyoma and lymphoma. The results of this study provide essential references for establishing imaging standards for preoperative diagnosis of breast leiomyoma, especially by filling the gap between elastic imaging and CEUS data in the existing literature.

Preoperative diagnosis of breast masses relies on multiple imaging modalities. MMG demonstrates specificity in detecting microcalcifications but insufficient sensitivity for lesions ≤1 cm and faces significant limitations in dense breasts (19, 20). Ultrasound is the primary imaging modality (21), and grayscale US visualizes morphological features (22). Color Doppler flow imaging can directly display the distribution of blood flow within and around the mass. However, the sensitivity of this technique in detecting blood flow is easily affected by various human operational factors, such as probe pressure and adjustment of the sound beam angle. Moreover, its ability to detect tiny blood vessels with a diameter of ≤ 0.2mm (especially low-flow vessels) is significantly reduced (23) (24). Both techniques are widely employed for breast mass evaluation, but their diagnostic specificity in distinguishing benign from malignant lesions remains limited (25). SWE and SE enable quantification of tissue stiffness, with studies demonstrating superior sensitivity and specificity compared with conventional US (26). CEUS utilizes approximately 2.5 μm microbubble contrast agents to visualize tumoral microcirculation (27). CEUS facilitates benign-malignant differentiation through image analysis: Benign features include well-defined margins, non-enhancement or homogeneous centripetal enhancement, absence of enlarged enhancement area, and arborizing vascular patterns. Malignant CEUS manifests as heterogeneous hyperenhancement with enlarged enhancement areas and peripheral crab claw-like infiltrations (28–30). Regarding quantitative parameters, CEUS metrics derived from time-intensity curves (AT, TTP, PI) have proven valuable for differentiation, with malignant lesions characteristically showing significantly shortened TTP and elevated PI (28, 31, 32). Previous studies have shown that contrast-enhanced ultrasound (CEUS) and elastography, used as single modalities, have high diagnostic value. For instance, a 2022 meta-analysis conducted by Wang et al. demonstrated the value of CEUS in differentiating benign from malignant conditions (33); at the same time, the research by Rafia Shahzad et al. also affirmed the significant advantages of elastography as a supplementary means to conventional ultrasound (34). In recent years, studies have increasingly emphasized the diagnostic enhancement value of multimodal combined applications. Chen et al.’s prospective study published in 2022 confirmed that the multimodal scheme combining conventional ultrasound, elastography, and CEUS has significantly better comprehensive diagnostic efficacy than any single mode (25). It is worth noting that Li et al.’s 2025 study further focused on the diagnostic difficulty of BI-RADS category 4, and the results showed that the combined use of CEUS and elastography could significantly improve the sensitivity and specificity of diagnosis (35).

In this case, CEUS revealed heterogeneous hyperenhancement of the lesion, but it also exhibited two critical features: well-defined margins and no expansion of the enhancement area, leading to an overall risk classification that could still be considered benign (36). However, the irregular shape, uneven internal echoes, and posterior acoustic shadowing observed on grayscale ultrasound, along with the relatively firm consistency indicated by elastography, were all definitive features suggestive of malignancy. By integrating these contradictory imaging findings, the mass was classified as BI-RADS 4b (moderately suspicious).

Breast leiomyoma is extremely rare, and in clinical practice, when encountering a breast mass with such characteristics, it is crucial to construct a systematic differential diagnosis framework. The mass described in this article has features such as “clear boundaries but irregular shape”, “coarse internal calcification”, “abundant blood flow signals”, “somewhat hard elasticity”, and “uneven high enhancement but no expansion of the enhancement range” on contrast imaging, making it difficult to distinguish from several more common breast tumors, and thus becoming the focus of clinical decision-making. Breast fibroadenomas usually have clear boundaries, regular shape, soft elasticity, and present benign contrast patterns (4, 37). The morphological irregularity, uneven internal echoes, large calcifications and abundant blood flow signals in this case do not match those of a typical fibroadenoma. Breast leiomyosarcoma is extremely rare, and its imaging features (such as rich blood supply and hard, solid masses) often overlap with those of leiomyomas, making imaging differentiation difficult (38). The gold standard for differentiating between the two relies on pathological examination, especially the assessment of mitotic counts, cellular atypia, and the presence or absence of necrosis (38). At the same time, it is also necessary to distinguish it from breast cancer (39), breast lymphoma (40), etc., which present as unclear boundaries, irregular shapes, sand-grain-like calcifications, elastic imaging indicating a relatively hard texture, and other malignant tumors as shown by malignant ultrasound contrast.

This case highlights a significant diagnostic pitfall. The initial core needle biopsy (CNB) yielded a diagnosis of “adenosis with focal fibroadenomatoid changes,” which was markedly discordant with the final surgical pathology of leiomyoma. A postoperative multidisciplinary review concluded that this discrepancy was primarily due to sampling error, likely attributable to the lesion’s relatively large size and firm consistency (41) (42). The extreme tissue stiffness may have caused the biopsy needle to deflect or sample only the peripheral compressed tissue, missing the diagnostic core. This experience underscores a crucial clinical lesson: for large, firm breast masses with atypical sonographic features, a benign CNB result must be interpreted with extreme caution, and surgical excision is often warranted for definitive diagnosis.

Therefore, histopathological examination, especially the assessment of the completely resected specimens, is the ultimate gold standard for diagnosis (43): the typical manifestation is a bundle of well-defined spindle-shaped cells with elongated cigar-shaped nuclei and bluntly rounded ends, with cytoplasmic eosinophilic staining evident and characteristic perinuclear vacuolization in the majority of cases. Interstitial changes included varying degrees of collagen fiber interlacing and focal fibrosis. Immunohistochemical confirmation using SMA and junctional proteins was diagnostic (43). However, the large amount of collagen fibres deposited in it reduces the tissue’s ability to deform and increases its density and wave velocity. This might be why the elastic imaging results in this case of breast leiomyoma are relatively complex (44).

Complete surgical resection is the preferred and usually curative treatment for this disease (45, 46). Among the published cases, except for two cases reported by Boscaino et al. where the initial diagnosis was leiomyoma but the lesion showed increased mitotic activity after histological re-examination and was reclassified as “uncertain prognosis leiomyoma”, and these cases had local recurrence (47), all the other reported cases showed no recurrence after complete resection (12). Therefore, if tumor recurrence occurs after surgery, it is necessary to pay close attention and re-evaluate to rule out the possibility of leiomyosarcoma. Based on this, it is recommended that patients with this disease undergo regular postoperative follow-up to monitor their condition.

Beyond the clear diagnosis and treatment principles, the unique comorbidity phenomenon of “follicular lymphoma combined with breast leiomyoma” in this case provides clues for exploring its underlying pathogenesis. In 2025, Loreta Canivilo Salas et al. demonstrated that BCL6 is involved in the pathogenesis of uterine leiomyoma, potentially promoting aberrant smooth muscle cell proliferation (48). It is noteworthy that in this case of a patient with follicular lymphoma, the lymphoma specimen was found to be BCL6-positive. However, since the BCL6 immunohistochemical test was not performed on the breast leiomyoma tissue in this case, it remains to be further confirmed by future studies whether the BCL6 signalling pathway plays a role in the occurrence and development of breast leiomyoma. The development of breast leiomyoma is still closely related to sex hormone regulation. Their growth may be influenced by signalling pathways involving estrogen and progesterone receptors (49). Some case reports suggest that long-term tamoxifen treatment may be potentially associated with the development of this tumor (50). Still, the causal relationship between the R-CHOP chemotherapy regimen (containing rituximab, cyclophosphamide, prednisone acetate tablets, and other medications) used in this case and the breast leiomyoma has not been supported in the literature, and further studies are needed to confirm it.

## Conclusion

The rarity of breast leiomyoma poses a significant challenge to preoperative imaging diagnosis. This article reports a rare case of breast leiomyoma combined with lymphoma, detailing its preoperative imaging features and integrating a literature review. We anticipate that in the future, more preoperative imaging reports of breast leiomyoma will be accumulated, and when a sufficient sample size is reached, it will help to refine the characteristic imaging patterns of this lesion, thereby improving diagnostic accuracy and optimizing clinical decisions, and ultimately improving patient prognosis.

## Data Availability

The raw data supporting the conclusions of this article will be made available by the authors, without undue reservation.

## References

[1] Kaufman HL Hirsch EF . Leiomyoma of the breast. J Surg Oncol. (1996) 62:62–4. doi: 10.1002/(SICI)1096-9098(199605)62:1<62::AID-JSO13>3.0.CO;2-V, PMID: 8618404

[2] Ende L Mercado C Axelrod D Darvishian F Levine P Cangiarella J . Intraparenchymal leiomyoma of the breast: a case report and review of the literature. Ann Clin Lab Sci. (2007) 37:268–73. https://pubmed.ncbi.nlm.nih.gov/17709693/., PMID: 17709693

[3] Strong LW . Leiomyoma of the breast. Am J Obstet. (1913) 68:53–5.

[4] Diaz-Arias AA Hurt MA Loy TS Seeger RM Bickel JT . Leiomyoma of the breast. Hum Pathol. (1989) 20:396–9. doi: 10.1016/0046-8177(89)90052-x, PMID: 2467872

[5] Kotsuma Y Wakasa K Yayoi E Kishibuchi M Kishibuchi M Sakamoto G . A case of leiomyoma of the breast. Breast Cancer. (2001) 8:166–9. doi: 10.1007/BF02967498, PMID: 11342992

[6] Kafadar MT Yalcin M Gok MA Aktas A Yurekli TS Arslan AI . Intraparenchymal leiomyoma of the breast: a rare location for an infrequent tumor. Eur J Breast Health. (2017) 13:156–8. doi: 10.5152/ejbh.2017.3472, PMID: 28894856 PMC5544138

[7] Arrob A Sabah TN AbouChadi A . Leiomyoma in breast ectopic tissue. Presse Med. (2019) 48:1587–8. doi: 10.1016/j.lpm.2019.09.037, PMID: 31759791

[8] Craig JM . Leiomyoma of the female breast. Arch Pathol (Chic). (1947) 44:314–7. Available online at: https://pubmed.ncbi.nlm.nih.gov/20267698/., PMID: 20267698

[9] Granic M Stefanovic-Radovic M Zdravkovic D Ivanovic N Nikolic D Radovanovic D . Intraparenchimal leiomyoma of the breast. Arch Iran Med. (2015) 18:608–12. Available online at: https://pubmed.ncbi.nlm.nih.gov/26317604/., PMID: 26317604

[10] Sampaio GP Koch MV Boechat M Matos VE Dos Santos AA . Leiomyoma of the breast: an uncommon tumor. Radiol Bras. (2016) 49:343–4. doi: 10.1590/0100-3984.2014.0136, PMID: 27818553 PMC5094828

[11] Kafadar MT Yalçın M Gök MA Aktaş A Yürekli TS Arslan Aİ . Intraparenchymal leiomyoma of the breast: A rare location for an infrequent tumor. Eur J Breast Health. (2017) 13:156–8. doi: 10.5152/ejbh.2017.3472, PMID: 28894856 PMC5544138

[12] Brandão RG Elias S Pinto Nazário AC Alcoforado Assunção MDCG Esposito Papa CC Facina G . Leiomyoma of the breast parenchyma: a case report and review of the literature. Sao Paulo Med J. (2017) 136:177–81. doi: 10.1590/1516-3180.2016.0253040117, PMID: 28977094 PMC9879555

[13] Yuksel ME Tamer F Bozlak N . Estrogen receptor positive, progesterone receptor negative, leiomyoma of the areola of a male patient. Dermatol Online J. (2018) 24:13030/qt82z7f5bz. doi: 10.5070/D3246040676, PMID: 30142715

[14] Zhong E Swistel A Viswanathan K Hoda SA . Leiomyoma of the Nipple: A common neoplasm in an uncommon location. Breast J. (2020) 26:529–30. doi: 10.1111/tbj.13572, PMID: 31486152

[15] Cai S Wang H Zhu Q Li J Sun Q Jiang Y . Clinical and sonographic features of nipple lesions. Med (Baltimore). (2020) 99:e19728. doi: 10.1097/MD.0000000000019728, PMID: 32282731 PMC7220069

[16] Chiorean A Pintican RM Szep M Feier D Rogojan L Fetica B . Nipple ultrasound: A pictorial essay. Korean J Radiol. (2020) 21:955–66. doi: 10.3348/kjr.2019.0831, PMID: 32677380 PMC7369201

[17] Zhang S Wang L Yang J Lu M . Ultrasound-guided microwave ablation for giant breast leiomyoma: A case report. Front Oncol. (2023) 13:1095891. doi: 10.3389/fonc.2023.1095891, PMID: 36741011 PMC9893409

[18] Raghunath P Maharaj R Harnanan D Barrow S Naraynsingh V . Leiomyoma of the breast: A report of a rare case of a benign breast tumor from the caribbean. Cureus. (2025) 17:e80678. doi: 10.7759/cureus.80678, PMID: 40236331 PMC11999726

[19] Sidoni A Lüthy L Bellezza G Consiglio MA Bucciarelli E . Leiomyoma of the breast: case report and review of the literature. Breast. (1999) 8:289–90. doi: 10.1054/brst.1999.0061, PMID: 14965748

[20] Bahl M Gadd MA Lehman CD . JOURNAL CLUB: diagnostic utility of MRI after negative or inconclusive mammography for the evaluation of pathologic nipple discharge. AJR Am J Roentgenol. (2017) 209:1404–10. doi: 10.2214/AJR.17.18139, PMID: 28898125

[21] Berg WA Bandos AI Mendelson EB Lehrer D Jong RA Pisano ED . Ultrasound as the primary screening test for breast cancer: analysis from ACRIN 6666. J Natl Cancer Inst. (2015) 108:djv367. doi: 10.1093/jnci/djv367, PMID: 26712110 PMC5943835

[22] Daly MB Pal T Berry MP Buys SS Dickson P Domchek SM . Genetic/familial high-risk assessment: breast, ovarian, and pancreatic, version 2.2021, NCCN clinical practice guidelines in oncology. J Natl Compr Canc Netw. (2021) 19:77–102. doi: 10.6004/jnccn.2021.0001, PMID: 33406487

[23] Raza S Baum JK . Solid breast lesions: evaluation with power Doppler US. Radiology. (1997) 203:164–8. doi: 10.1148/radiology.203.1.9122386, PMID: 9122386

[24] Yoo J Je BK Choo JY . Ultrasonographic demonstration of the tissue microvasculature in children: microvascular ultrasonography versus conventional color doppler. Korean J Radiol. (2020) 21:146–58. doi: 10.3348/kjr.2019.0500, PMID: 31997590 PMC6992447

[25] Chen Y Lu J Li J Liao J Huang X Zhang B . Evaluation of diagnostic efficacy of multimode ultrasound in BI-RADS 4 breast neoplasms and establishment of a predictive model. Front Oncol. (2022) 12:1053280. doi: 10.3389/fonc.2022.1053280, PMID: 36505867 PMC9730703

[26] Sigrist RMS Liau J Kaffas AE Chammas MC Willmann JK . Ultrasound elastography: review of techniques and clinical applications. Theranostics. (2017) 7:1303–29. doi: 10.7150/thno.18650, PMID: 28435467 PMC5399595

[27] Boca Bene I Dudea SM Ciurea AI . Contrast-enhanced ultrasonography in the diagnosis and treatment modulation of breast cancer. J Pers Med. (2021) 11:81. doi: 10.3390/jpm11020081, PMID: 33573122 PMC7912589

[28] Du J Wang L Wan CF Hua J Fang H Chen J . Differentiating benign from Malignant solid breast lesions: combined utility of conventional ultrasound and contrast-enhanced ultrasound in comparison with magnetic resonance imaging. Eur J Radiol. (2012) 81:3890–9. doi: 10.1016/j.ejrad.2012.09.004, PMID: 23062280

[29] Xiao X Ou B Yang H Wu H Luo B . Breast contrast-enhanced ultrasound: is a scoring system feasible? A preliminary study in China. PloS One. (2014) 9:e105517. doi: 10.1371/journal.pone.0105517, PMID: 25133534 PMC4136879

[30] Li CY Gong HY Ling LJ Du LW Su T Wang S . Diagnostic performance of contrast-enhanced ultrasound and enhanced magnetic resonance for breast nodules. J BioMed Res. (2018) 32:198–207. doi: 10.7555/JBR.32.20180015, PMID: 29921747 PMC6103473

[31] Nakata N Ohta T Nishioka M Takeyama H Toriumi Y Kato K . Optimization of region of interest drawing for quantitative analysis: differentiation between benign and Malignant breast lesions on contrast-enhanced sonography. J Ultrasound Med. (2015) 34:1969–76. doi: 10.7863/ultra.14.10042, PMID: 26384607

[32] Wan C Du J Fang H Li F Wang L . Evaluation of breast lesions by contrast enhanced ultrasound: qualitative and quantitative analysis. Eur J Radiol. (2012) 81:e444–50. doi: 10.1016/j.ejrad.2011.03.094, PMID: 21612882

[33] Wang J Zhao R Cheng J . Diagnostic accuracy of contrast-enhanced ultrasound to differentiate benign and Malignant breast lesions: A systematic review and meta-analysis. Eur J Radiol. (2022) 149:110219. doi: 10.1016/j.ejrad.2022.110219, PMID: 35228171

[34] Shahzad R Fatima I Anjum T Shahid A . Diagnostic value of strain elastography and shear wave elastography in differentiating benign and Malignant breast lesions. Ann Saudi Med. (2022) 42:319–26. doi: 10.5144/0256-4947.2022.319, PMID: 36252146 PMC9557788

[35] Li C Wu M Haiqing H . The application of multimodal ultrasound examination in the differential diagnosis of benign and Malignant breast lesions of BI-RADS category 4. Front Med (Lausanne). (2025) 12:1596100. doi: 10.3389/fmed.2025.1596100, PMID: 40552183 PMC12183244

[36] Xiao X Dong L Jiang Q Guan X Wu H Luo B . Incorporating contrast-enhanced ultrasound into the BI-RADS scoring system improves accuracy in breast tumor diagnosis: A preliminary study in China. Ultrasound Med Biol. (2016) 42:2630–8. doi: 10.1016/j.ultrasmedbio.2016.07.005, PMID: 27544439

[37] Son EJ Oh KK Kim EK Son HJ Jung WH Lee HD . Leiomyoma of the breast in a 50-year-old woman receiving tamoxifen. AJR Am J Roentgenol. (1998) 171:1684–6. doi: 10.2214/ajr.171.6.9843313, PMID: 9843313

[38] Kim BR Lee JH Cho E Kim DC Park YM Ha DH . Primary breast leiomyosarcoma located in the premammary zone: a case report. Clin Imaging. (2015) 39:1105–7. doi: 10.1016/j.clinimag.2015.06.014, PMID: 26278012

[39] Cao XL Bao W Zhu SG Wang LH Sun MH Wang L . Contrast-enhanced ultrasound characteristics of breast cancer: correlation with prognostic factors. Ultrasound Med Biol. (2014) 40:11–7. doi: 10.1016/j.ultrasmedbio.2013.08.014, PMID: 24207087

[40] Chen W Liu F Wang R Qi M Zhang J Liu X . End-to-end deep learning radiomics: development and validation of a novel attention-based aggregate convolutional neural network to distinguish breast diffuse large B-cell lymphoma from breast invasive ductal carcinoma. Quant Imaging Med Surg. (2023) 13:6598–614. doi: 10.21037/qims-22-1333, PMID: 37869296 PMC10585556

[41] Huang XC Hu XH Wang XR Zhou CX Wang FF Yang S . A comparison of diagnostic performance of vacuum-assisted biopsy and core needle biopsy for breast microcalcification: a systematic review and meta-analysis. Ir J Med Sci. (2018) 187:999–1008. doi: 10.1007/s11845-018-1781-6, PMID: 29549564

[42] Cavaliere A Sidoni A Scheibel M Bellezza G Brachelente G Vitali R . Biopathologic profile of breast cancer core biopsy: is it always a valid method? Cancer Lett. (2005) 218:117–21. doi: 10.1016/j.canlet.2004.07.041, PMID: 15639347

[43] Dhankar N Tomar R Paul S Khurana N Neogi S . Leiomyoma of the nipple: A rare entity at rare site. Cureus. (2024) 16:e62220. doi: 10.7759/cureus.62220, PMID: 39006682 PMC11240242

[44] Obrzut M Obrzut B Zmuda M Baran J Cholewa M Ehman R . Uterine leiomyomas: correlation between histologic composition and stiffness via magnetic resonance elastography - a Pilot Study. Ginekol Pol. (2020) 91:373–8. doi: 10.5603/GP.a2020.0067, PMID: 32542642 PMC7958487

[45] Lauwers G de Roux S Terzakis J . Leiomyoma of the breast. Arch Anat Cytol Pathol. (1990) 38:108–10. Available online at: https://pubmed.ncbi.nlm.nih.gov/2363589/., PMID: 2363589

[46] Shah SD Gupta A Roy S Mukhopadhyay S . Intraparenchymal leiomyoma of the breast: a case report. Indian J Surg. (2013) 75:88–9. doi: 10.1007/s12262-011-0367-6, PMID: 24426524 PMC3693225

[47] Pourbagher A Pourbagher MA Bal N Oguzkurt L Ezer A . Leiomyoma of the breast parenchyma. AJR Am J Roentgenol. (2005) 185:1595–7. doi: 10.2214/AJR.04.1453, PMID: 16304020

[48] Salas LC Mielczarski B Rivero RC da Cunha Filho JSL Savaris RF . BCL6 (B-cell lymphoma 6) expression in adenomyosis, leiomyomas and normal myometrium. PloS One. (2025) 20:e0317136. doi: 10.1371/journal.pone.0317136, PMID: 39903727 PMC11793761

[49] Hammer P White K Mengden S Korcheva V Raess PW . Nipple leiomyoma: A rare neoplasm with a broad spectrum of histologic appearances. J Cutan Pathol. (2019) 46:343–6. doi: 10.1111/cup.13423, PMID: 30663114

[50] Bayya J Minkoff H Khulpateea N . Tamoxifen and growth of an extrauterine leiomyoma. Eur J Obstet Gynecol Reprod Biol. (2008) 141:90–1. doi: 10.1016/j.ejogrb.2008.07.001, PMID: 18848745

